# Regulation of Interfacial Anchoring Orientation of Anisotropic Nanodumbbells

**DOI:** 10.1021/acsmacrolett.3c00339

**Published:** 2023-09-11

**Authors:** Hyunwoo Jang, Chaeyeon Song, Byungsoo Kim, Chunghyeong Lee, Juncheol Lee, Youngkyu Han, Ilsin An, Joon Heon Kim, Jin Nam, Myung Chul Choi

**Affiliations:** † Department of Bio and Brain Engineering, Korea Advanced Institute of Science and Technology, Daejeon 34141, South Korea; ‡ AMOREPACIFIC R&I Center, Yongin 17074, South Korea; § Department of Photonics and Nanoelectronics, Hanyang University, Ansan 15588, South Korea; ∥ Advanced Photonics Research Institute, Gwangju Institute of Science and Technology, Gwangju 61005, South Korea

## Abstract

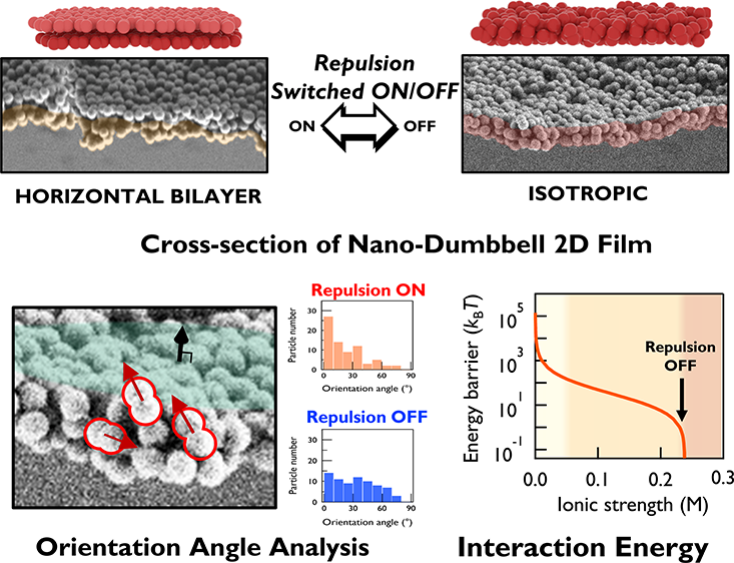

Nanoparticles exhibiting geometrical and chemical anisotropies hold promise for environmentally responsive materials with tunable mechanical properties. However, a comprehensive understanding of their interfacial behaviors remains elusive. In this paper, we control the interfacial anchoring orientation of polystyrene nanodumbbells by adjusting interparticle forces. The film nanostructure is characterized by the orientation angle analysis of individual dumbbells from cross-sectional EM data: dumbbells undergo orientation transitions from a distinctive horizontal bilayer to an isotropic anchoring when electrostatic repulsion is suppressed by either an ionic strength increase or surface amine-modification. This anchoring orientation influences the film’s mechanical properties and foam stability, as investigated by a 2D isotherm and dark/bright-field microscopy measurements. Our findings highlight the potential for precise control of supra-colloidal structures by modulating particle alignment, paving the way for smart delivery systems.

Pickering stabilization, a phenomenon where solid nanoparticles (NPs) stabilize a fluid–fluid interface, has been utilized in drug delivery,^1,2^ catalysis,^3^ cosmetics,^4^ and food engineering.^5^ NPs exhibit adhesion energy significantly higher than that of molecular surfactant and thus bind to interfaces nearly irreversibly. By this, they serve as steric barriers^6,7^ and make the interface highly resistant to coalescence.^6,8,9^ Another unique advantage of NPs is their programmability: their geometry and chemical properties can be tailored throughout synthesis and modification processes, often anisotropically.^10−12^

Recent research has emphasized the impact of geometrical and chemical anisotropy of NPs on their interfacial behaviors.^11,13,14^ The shape and surface chemistry (i.e., charge and hydrophilicity) of anisotropic nanoparticles (ANPs) collectively orchestrate their interfacial characteristics, by influencing the trapping energy, equilibrium orientation, and 2D packing structures.^7,9,11,15−18^

However, most existing studies have primarily focused on individual particle level.^11^ Also, strategies to control the behaviors of a particle with given anisotropy profiles remain underexplored. Adding another dimension of complexity, the modulation of “supracolloidal” level characteristics (i.e., 2D film and foam formation) of ANPs by tuning the underlying interparticle forces, can provide valuable insights for a comprehensive understanding of the interfacial properties of ANPs. This knowledge can also be used to leverage the stability and programmability of ANPs in emulsification and delivery systems.

Herein, we investigate the effect of interparticle forces on the interfacial behavior and film formation of polystyrene (PS) nanodumbbells. Dumbbell shape was chosen due to its versatility in controlling the size ratio and surface chemistry of each lobe.^19−21^ Bulk-scale synthesis with uniform size distribution has also been achieved.^22−24^ We hypothesize that changes in interparticle force would alter the interfacial orientation of nanodumbbells and subsequently modulate the mechanical properties of ANP films.

Using two-step emulsion polymerization technique,^22,23,25,26^ we synthesized symmetric nanodumbbells (denoted *db*_1_, Figure 1A,C) from sulfonate-stabilized polystyrene spheres (*sp*; Figure 1A,B). A *db*_1_ particle has two equal-sized lobes: a seed lobe (originally core–shell seed) and a budded lobe. Using an amino-silane coupling agent, we amine-modified the seed lobes of *db*_1_ and obtained charge-anisotropic Janus dumbbells (denoted as *db*_2_, Figure 1A,D). Localization of amine groups on the seed lobes was confirmed by the selective adsorption of anionic gold nanoparticles (AuNPs; inset of Figure 1D and Figure S1). Using the Grahame equation and zeta potential values (Figure 1E), the charge densities (σ) of each NP and lobe were determined (Figure 1F, Table 1, and SI text).^27,28^

**Table 1:** Size Parameters and Charge Characteristics of NPs^a^

particle	*R* (nm)	*L* (nm)	aspect ratio	*A*_CP_ (10^4^ nm^2^)	charge density at pH 7.5 (*e* nm^–2^)
*sp*	67 ± 2		1	1.56	–0.36
*db*_1_	85 ± 2	297 ± 9	1.74 ± 0.05	4.55	–0.23 (BL: −0.29; SL: −0.17)
*db*_2_	84 ± 4	291 ± 8	1.74 ± 0.08	4.36	–0.13 (BL: −0.29; SL: +0.03)

a *R*: radius of the host sphere; *L*: length of the dumbbell. Errors are standard deviations (*n* = 30). *A*_CP_: close packing area. The *A*_CP_ values of dumbbells correspond to a horizontal orientation. BL: budded lobe. SL: seed lobe.

**Figure 1:**
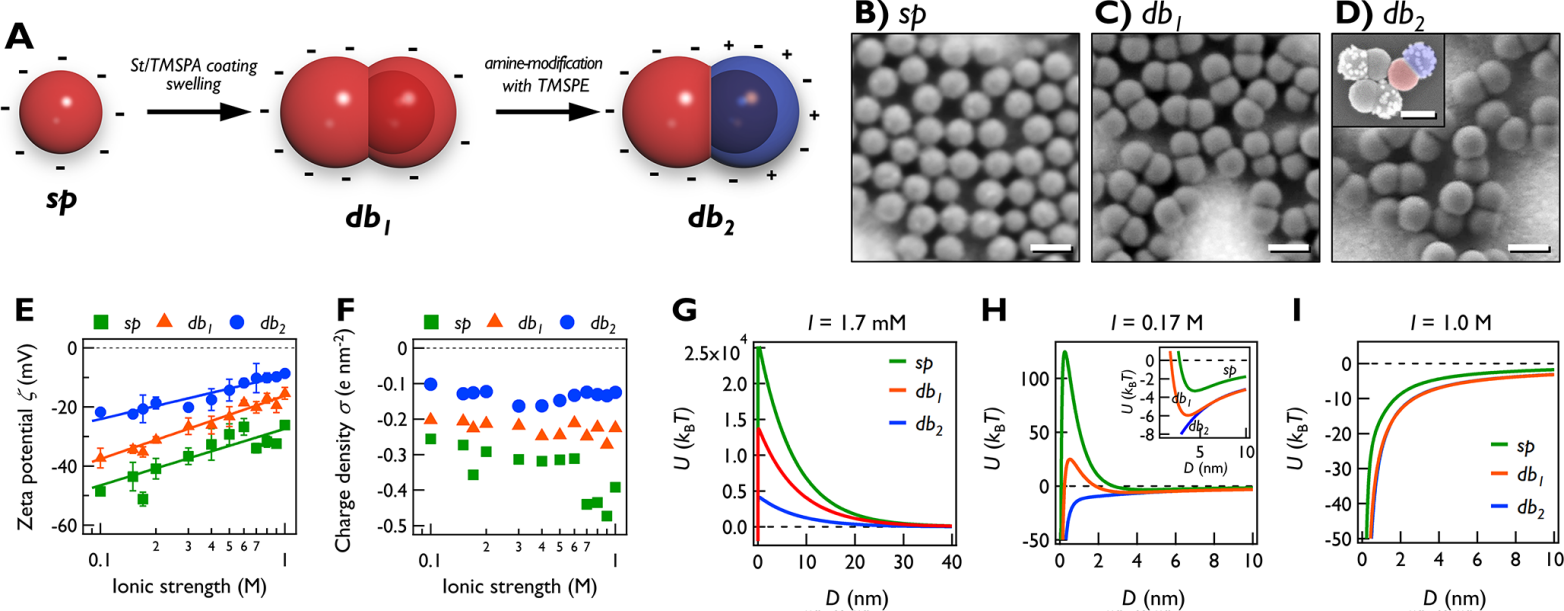
Nanoparticle synthesis and characterization. (A) Schematic illustration of ANP synthesis by two-step seeded emulsion polymerization and selective amine-modification. The numbers of charges are in scale. (B–D) Representative SEM images of (B) *sp*, (C) *db*_1_, and (D) *db*_2_ (inset of D: negatively charged AuNPs adsorbed on amine-modified seed lobes of *db*_2_). Scale bars: 200 nm. (E) Zeta potential and (F) effective charge density of three NPs vs ionic strength at pH ≈ 7.5. Solid lines: logarithmic fits. (G–I) Pair interaction potential energy (*U*) of three NPs vs surface-to-surface distance *D* at *I* = (G) 1.7 mM, (H) 0.17 M, and (I) 1.0 M. The curves of *db*_1_ and *db*_2_ are the average of all approaching directions and relative orientations. In part I, the curves of *db*_1_ and *db*_2_ are nearly overlapped.

Based on the shape and charge profiles, we calculated pairwise potential energy of the three NPs anchored at the air–water interface as the sum of van der Waals attraction and electrostatic repulsion (*U* = *V*_vdW_ + *V*_el_; Figure 1G–I).^28,29^ At *I* = 1.7 mM, long-range electrostatic repulsion dominates, resulting in a high (∼10^4^*k*_B_*T*) energy barrier (*U*_barrier_; Figure 1G). The repulsion of dumbbells is weaker than *sp*. For *db*_1_, interparticle interactions are repulsive for all approaching directions, hindering particle attachment (Figure S2). However, for *db*_2_, although the average energy is repulsive, attraction occurs in ∼50% of the approaching directions (*U* ≈ –3 × 10^3^*k*_B_*T* at surface-to-surface distance *D* = 3 nm; Figure S3), suggesting the possibility of *db*_2_ clustering even at low salt.

At *I* = 0.17 M, the electrostatic repulsion is reduced (Figure 1H). For *sp* and *db*_1_, *U*_barrier_ decreases to 125 and 25*k*_B_*T*, respectively, with equilibrium distances *D*_eq_ (*D* at the secondary minimum) of 4.4 and 3.7 nm. This implies that two distant particles will be drawn together only until they reach an equilibrium spacing at *D*_eq_. In the case of *db*_2_, attraction is dominant and secondary minimum is absent. At higher *I*, the *U*_barrier_ values of *db*_1_ and *sp* subsequently decreased and became zero at *I* = 0.24 M (*db*_1_) and 0.46 M (*sp*; Figure S4). At *I* = 1.0 M, repulsion is almost completely suppressed, and attraction becomes dominant for all three particles (Figure 1I), suggesting that particles would strongly flocculate.

We examined the foaming ability of the NPs (Figure 2A,B). Typical anionic PS latex shows poor foaming due to electrostatic repulsion.^30^ Our *sp* particles also failed to form foams even at *I* = 1.0 M (Figure S5), while both nanodumbbells successfully stabilized foams. While *db*_2_ formed foams even at *I* = 0 M (Figure 2B), the foaming ability of *db*_1_ varied dramatically with ionic strength. While no foams were seen at *I* < 0.1 M, unstable (lifetime of ∼24 h) foams appeared at *I* = 0.17 M. This instability may be due to their equilibrium spacing, making the foams “leaky”. Under higher *I* (≥0.25 M), *db*_1_ stabilized foams as effectively as *db*_2_. These dumbbell-stabilized stable foams exhibited remarkable resistance, lasting over one month.

**Figure 2:**
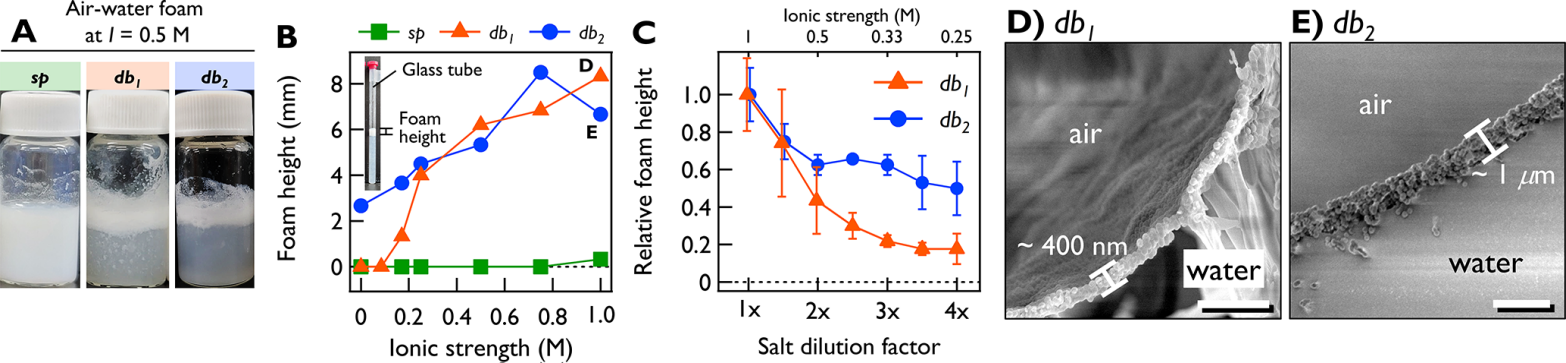
Ion-dependent foaming and defoaming by ANPs. (A) Dumbbell-stabilized air–water foams at *I* = 0.5 M 1 h after foaming. (B) Foam height measured in glass tubes (inset) 10 min after foaming as a function of ionic strength. (C) Defoaming of *db*_1_ and *db*_2_ by salt dilution (initial *I* = 1.0 M). Corresponding ionic strength is denoted at the top axis. Cryo-SEM images of foam cross sections of (D) *db*_1_ and (E) *db*_2_ at *I* = 1.0 M. Scale bars: 2 μm.

Figure 2C quantifies defoaming due to ionic strength decrease. At 0.25 M, approximately half of *db*_2_ foams remained, while *db*_1_ foams disappeared to a greater extent (>80%). This ion-robustness of *db*_2_ foams agrees with their interparticle attraction in a wider ionic regime. We examined the cross-section of dumbbell-stabilized foams with cryo-SEM (Figure 2D,E). The shell thickness was highly uniform (∼400 nm for *db*_1_ and ∼1 μm for *db*_2_). While spherical PS latex typically forms monolayer on foams,^30,31^ our dumbbell-stabilized foams were bi- or multilayers, which explains their structural robustness.

We investigated the internal structure of the NP films from their cross sections at the planar interface. Figure 3 shows cross-sectional SEM images of the Langmuir–Schaefer films transferred at π_c_ (onset pressure of the collapse phase, i.e., the highest pressure within the condensed phase; see Figure 4E for isotherm curves). In such highly compressed conditions, the film’s structural characteristics are preserved even after transferring.^32,33^ As in foam cross sections, both *db*_1_ and *db*_2_ films were uniform in thickness (400–500 nm). The *sp* formed a well-defined monolayer at *I* = 0.17 M and a bilayer or trilayer at *I* = 1.0 M (Figure S6).

**Figure 3:**
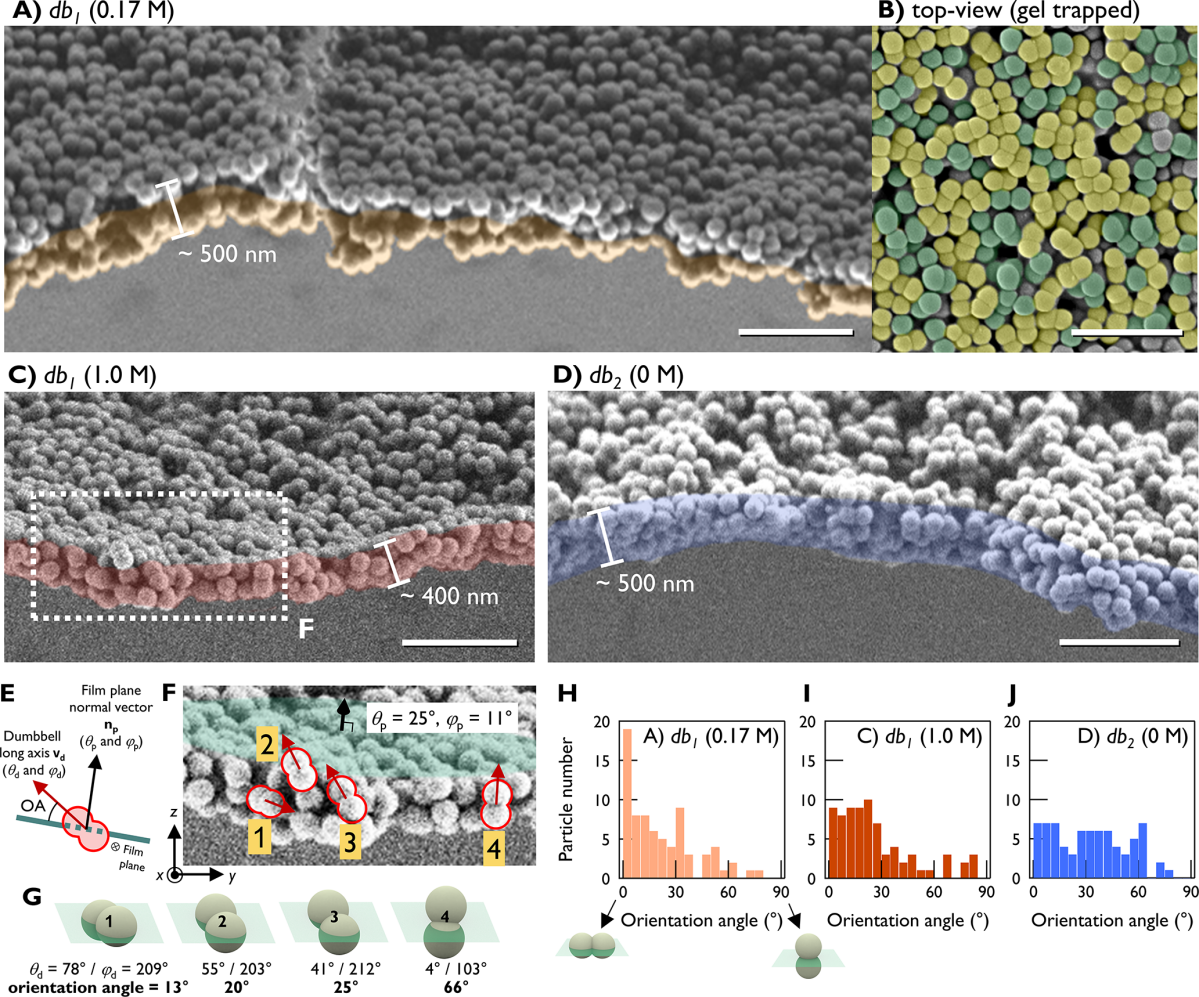
Anchoring structures of ANPs in the films. (A) Cross-section of *db*_1_ film at *I* = 0.17 M. Horizontally oriented *db*_1_ particles form two layers. Bottom layer is colored orange. (B) Cryo-SEM top-view image of a gel-trapped *db*_1_ film at 0.17 M. *db*_1_ with horizontal anchoring colored yellow, others green. (C) Cross-section of the *db*_1_ film at 1.0 M. (D) Cross-section of *db*_2_ film (*I* = 0 M). Scale bars: 1 μm. (E) Schematics of orientation angle (OA) of an ANP relative to the film plane. (F) Zoom-in of (C). Film plane and the normal vector angles (θ_p_ and φ_p_) are depicted. Four representative dumbbell particles are highlighted in red. (G) Schematics of the dumbbell particles in (F) embedded in the film plane. Dumbbell vector angles (θ_d_ and φ_d_) and orientation angles (OA) are depicted. OA distribution plots (*n* = 74) are (H) *db*_1_ at 0.17 M, (I) *db*_1_ at 1.0 M, and (J) *db*_2_ at 0 M. OA = 0° (horizontal) and 90° (vertical anchoring) depicted with schematics.

**Figure 4:**
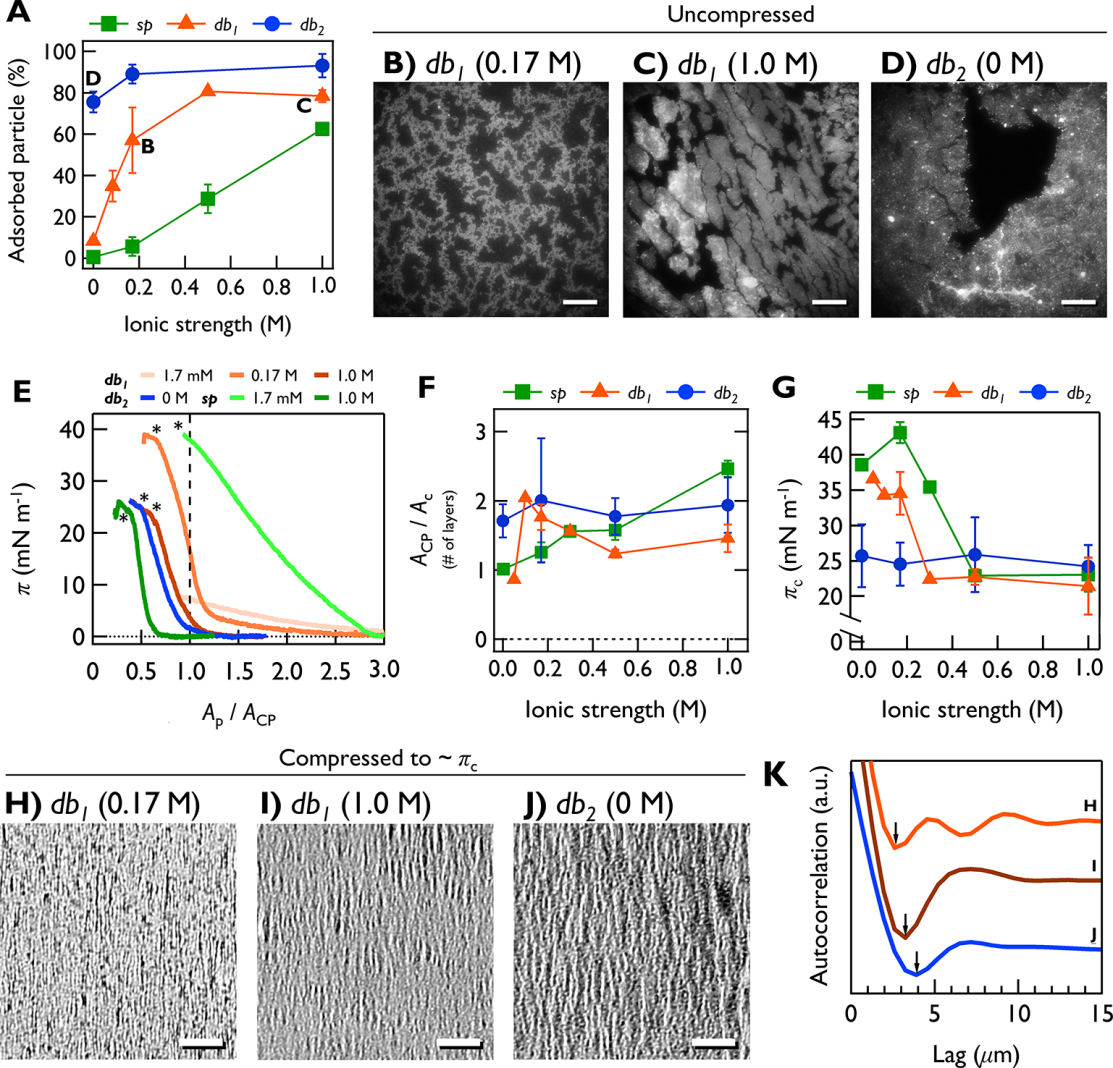
2D phase behaviors of the NP films. (A) Ion-regulated interfacial adsorption of NPs. NP adsorption was quantified by measuring subphase turbidity after deposition. Dark-field microscopy (DFM) images were taken for (B) *db*_*1*_ at 0.17 M, (C) *db*_1_ at 1.0 M, and (D) *db*_2_ at 0 M. Images were obtained before lateral compression (π = 0 mN m^–1^). ANPs in (C) and (D) are highly clustered. Scale bars: 200 μm. (E) Surface pressure–area per particle (*A*_p_) isotherm curves. *A*_p_ (*x*-axis) is normalized by the close-packing area (*A*_CP_). Collapse points are indicated by asterisks. (F) Ratio of *A*_CP_ and collapse area *A*_c_ showing the effective number of layers. (G) Collapse pressure π_c_ as a function of subphase ionic strength. Bright-field microscopy (BFM) images of (H) *db*_1_ at 0.17 M, (I) *db*_1_ at 1.0 M, and (J) *db*_2_, showing wrinkle phase of ANP films. Scale bars: 100 μm. (K) 1-D autocorrelation of (H), (I), and (J). First minima are indicated with arrows.

Notably, at *I* = 0.17 M, *db*_1_ forms a horizontal bilayer, in which dumbbells are oriented horizontally as two separate monolayers, and those two monolayers are stacked (Figure 3A: the bottom layer is colored orange; note the gap between the two layers). To confirm the dumbbells’ horizontal alignment, we trapped the particles in a polyacrylamide gel in situ and obtained top-view SEM images (Figure 3B). We could identify the majority of *db*_1_ particles aligned horizontally (colored yellow). In contrast, visual inspection showed that dumbbells in both *db*_1_ film at 1.0 M (Figure 3C) and *db*_2_ film at 0 M (Figure 3D) exhibited a wider range of orientation angle. Although horizontal particles were still present, a significant portion of the particles anchored nonhorizontally.

For a precise analysis, we extracted the orientation angle (OA; angle between the film plane and a dumbbell’s long axis) of individual dumbbells from cross-sectional SEM images. Briefly, the angles defining the 3D directions of the plane film’s normal vector (θ_p_ and φ_p_) and a dumbbell’s long axis vector (θ_d_ and φ_d_) were determined. Then we obtained the angle between the two vectors (Figure 3E–G; see SI text, Figures S7 and S8). Figure 3H–J plots the OA distributions of the dumbbells under three different conditions. For *db*_1_ at 0.17 M, the horizontal orientation is predominant, showing the highest occurrence at the 0–5° bin. When electrostatic repulsion is reduced by either ionic strength change or surface modification (*db*_1_ at 1.0 M and *db*_2_, respectively), the OA distributions widened, corroborating the aforementioned visual inspection. However, for all three conditions, horizontal anchoring (0–30°) was preferred to vertical anchoring (60–90°). This preference is due to the high (5.5 × 10^5^*k*_B_*T*) rotational energy barrier (Figure S9), which makes a dumbbell particle unlikely to rotate vertically if a particle is initially anchored horizontally upon deposition. Nonhorizontally oriented particles may have originated from particle clusters formed right after deposition. Since tight clustering is energetically stable, once stably clustered, particle orientation would be preserved throughout the lateral compression.

To elucidate the 2D interparticle interactions, we performed surface affinity and force measurements. Figure 4A shows the interfacial NP adsorption affinity (quantified from subphase turbidity after particle deposition) as a function of ionic strength. The *db*_2_ demonstrated robustly high surface affinity under all ion conditions. However, the adsorption affinity of *db*_1_ and *sp* showed dramatic increases from ∼0% to >60%, likely due to suppressed interparticle repulsion. The *db*_1_ surface affinity was higher than that of *sp*, consistent with stronger interparticle repulsion of *sp*.

Dark-field microscopy (DFM) revealed self-assembled cluster structures of nanodumbbells at zero surface pressure. The *db*_1_ cluster morphology was dependent on the ionic strength. At 0.17 M, *db*_1_ forms uniformly distributed 10 μm scaled clusters (Figure 4B). At 1.0 M, *db*_1_ clusters grew to submillimeter sizes and became polydisperse in thickness (greater variance in pixel brightness; Figure 4C). The *db*_2_ clusters were even larger (>1 mm) and also showed polydisperse thickness (Figure 4D). The *sp* clusters were not visible at 0.17 M but were observed at 1.0 M (Figure S10).

Figure 4E demonstrates the surface pressure–area per particle (π–*A*_p_) isotherm curves of the NPs. The *x*-axis was rescaled with interfacial adsorption affinity (accounting for particle submersion upon deposition) and close-packing area (*A*_CP_; Table 1). For each curve, the collapse area (*A*_c_) and the pressure (π_c_) were quantified. By calculating *A*_CP_/*A*_c_, we estimated the effective number of layers (Figure 4F). See Figure S11 for the entire isotherm curves.

The *db*_1_ isotherm curves transformed dramatically with changing *I*. At very low salt (≤2 mM), π increased monotonically up to <10 mN m^–1^, without sign of collapse. This suggests that *db*_1_ particles adsorbs only weakly and submerge upon compression.^30^ At 0.05 M, collapse occurs near *A*_CP_, suggesting the formation of monolayer. In the range of 0.1–0.2 M, the film thickness increases to bilayer (Figure 4F), which supports the occurrece of horizontal bilayer seen in Figure 3. The *db*_2_ isotherm did not change with *I* with a thickness corresponding to a bilayer under all ionic conditions. The *sp* film’s thickness showed a gradual increase from monolayer at 0 M to ∼2.5 layers at 1.0 M. This is in line with the cross-sectional SEM images in Figure 3.

Figure 4G illustrates the *I*-dependent changes in π_c_. The *db*_2_ displayed a highly constant (25 mN m^–1^) π_c_. In contrast, the π_c_ of *db*_1_ abruptly decreased from 35 to 21 mN m^–1^ at *I* = 0.3 M. The *sp* also showed a sudden decrease from 40 to 23 mN m^–1^ at *I* = 0.5 M. These thresholds align well with the energy barrier decrease (Figures 5A and S4). Under the presence of equilibrium distancing, the repulsive force might effectively dissipate the lateral compressive stress throughout the film. Therefore, the film might withstand stronger lateral compression, leading to higher π_c_. If attraction is dominant (*db*_1_ and *sp* at high salt or *db*_2_), particle clusters assembled right after deposition are jammed upon compression. Cluster–cluster boundary regions might be structurally weaker, making the entire film more heterogeneous in thickness and mechanical strength.^32^ When stress is concentrated at those weaker regions, the film may collapse at a lower π_c_. This π_c_ decrease may also explain the thinning of the *db*_1_ film from 2 to 1.5 layers at *I* ≥ 0.3 M. Because collapse occurs at a lower π_c_, the film thickness at *A*_c_ may also have decreased.

**Figure 5:**
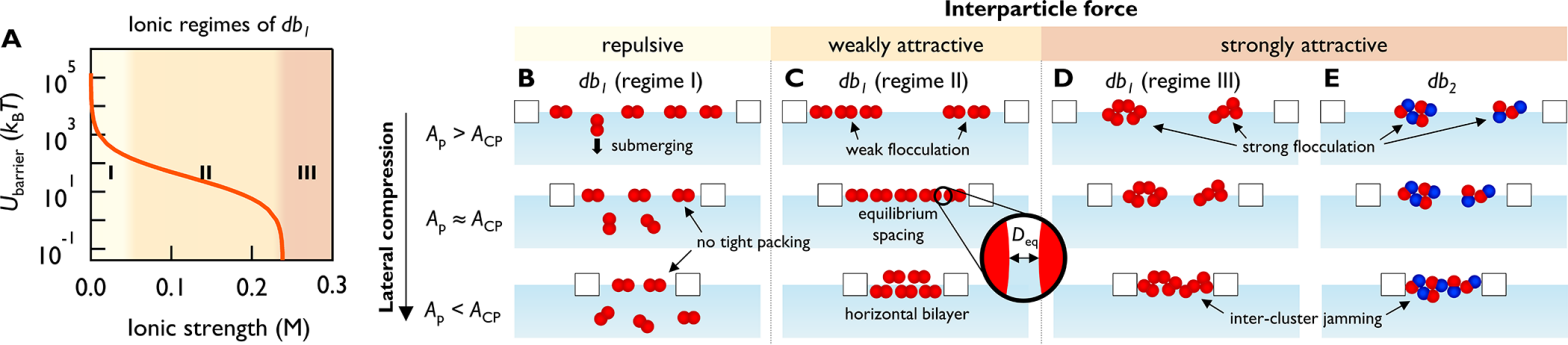
Schematic illustration of nanodumbbell film formation mechanisms. (A) Three ionic regimes of *db*_1_ and *U*_barrier_ (maximal energy in pair interaction potential). *U*_barrier_ of *db*_1_ reaches zero at *I* = 0.24 M. (B–E) Interfacial behaviors of nanodumbbells upon lateral compression at various interparticle force conditions. (B) At repulsive condition (*db*_1_ in regime I; *I* ≤ 0.02 M), dumbbells submerge upon compression and particle film is not formed due to strong repulsion. (C) At weakly attractive condition (*db*_1_ in regime II; *I* = 0.05–0.24 M), dumbbells form a monolayer with equilibrium spacing (*D*_eq_). When compressed below *A*_CP_, out-of-plane slipping occurs and a horizontal bilayer is formed. (D, E) At strongly attractive conditions ((D): *db*_1_ in regime III; *I* ≥ 0.24 M and (E) *db*_2_), dumbbells spontaneously form tight clusters. Upon compression, intercluster jamming occurs, resulting in an isotropic film.

Before reaching π_c_, NP films undergo a wrinkling phase transition, as revealed by bright-field microscopy (BFM; Figure 4H–J, Movie S1). For *db*_1_ at 0.17 M, three local minima are observed in the 1D autocorrelation curve (i.e., higher spatial coherence; Figure 4K). For the other two conditions, only the first minimum is identified, indicating more heterogeneous wrinkle pitch (i.e., lower spatial coherence). Consequently, the DFM, BFM, and π_c_ data consistently indicate that ANP clustering becomes heterogeneous when repulsion is reduced.

From 1D autocorrelation curves, we quantified the wrinkles’ spatial wavelengths (λ = 2 × *L*_1_^min^ [*L*_1_^min^: lag at first minimum]) as λ = 5.2 μm (*db*_1_ at 0.17 M), 6.5 μm (*db*_1_ at 1.0 M), and 7.8 μm (*db*_2_). From these values, the bending rigidity of the films was estimated. The total energy of a film on a fluid substrate is minimized when the bending energy and the substrate deformation energy are balanced.^34^ Thus, the relationship between the equilibrium wrinkle wavelength (λ) and the bending rigidity (*B*) is given as *B* = ρ*g*(λ/2π)^4^, where ρ is fluid density and *g* is the gravitational acceleration.^34^ The calculated *B* values are 1.12*k*_B_*T* (*db*_1_ at 0.17 M), 2.73 *k*_B_T (*db*_1_ at 1.0 M), and 5.66 *k*_B_*T* (*db*_2_). The horizontal bilayer structure of *db*_1_ at 0.17 M has relatively weak layer–layer interaction because most particles lie horizontally and therefore do not engage in interlayer anchoring (note the interlayer gap in Figure 3A). This may make the film more susceptible to bending. Moreover, as shown in energy calculations, *db*_1_ could exhibit equilibrium distancing instead of tight packing, which also accounts for its low rigidity. At higher salt, *db*_1_ particles become more isotropically (nonhorizontally) oriented, increasing the number of interlayer anchoring points, which can explain increased rigidity. The *db*_2_ film exhibits an even wider OA distribution, indicating greater rigidity. These demonstrate that interparticle energy profile and subsequent anchoring behavior of the ANPs can impact the film‘s mechanical properties.

From the energy calculation and experimental results, we could specify three interparticle force conditions of repulsive, weakly attractive, and strongly attractive, each corresponding to a specific ionic regime for *db*_1_ (Figure 5A). These force conditions subsequently govern the film forming mechanism of our nanodumbbells. The boundary between regimes I and II is 0.05 M, corresponding to the lowest ionic strength condition that allowed stable layer formation (Figure S11B). The boundary between regimes II and III is 0.24 M, the condition where *U*_barrier_ becomes zero and equilibrium distancing disappears. This is also where π_c_ abruptly decreases in the 2D isotherm and the foam formation ability of *db*_1_ becomes similar to that of *db*_2_.

At repulsive conditions (*db*_1_ in regime I), dumbbells exhibit low surface affinity and do not form foams or films (Figure 5B). Tight packing does not occur and particles submerge by overcoming the attachment energy barrier of 7.3 × 10^5^*k*_B_*T* (Figure S9). At weakly attractive conditions (*db*_1_ in regime II), dumbbells spontaneously approach up to the equilibrium distance (*D*_eq_), forming thin and weak clusters. Because vertical rotation is energetically unfavorable (Figure S9C), particles transition to a bilayer arrangement while maintaining horizontal anchoring, which we have termed the *horizontal bilayer*. This structure may be “leaky”, i.e., gaps may be present between individual particles, as evidenced by low foam stability. At strongly attractive conditions (*db*_1_ in regime III and *db*_2_), repulsion is greatly reduced, further enhancing spontaneous cluster assembly, which leads to a broader distribution of orientation angles. Upon lateral compression, intercluster jamming occurs, resulting in lower collapse pressure and higher bending rigidity.

We have demonstrated the ionic regulation of the interfacial 2D anchoring of dumbbell-shaped anisotropic nanoparticles, noting a horizontal-to-isotropic transition with reduced interparticle repulsion. This shift also changed the mechanical properties of the films. The systemic investigation and control of supra-colloidal level behaviors (i.e., interfacial orientation and film formation) of anisotropic nanodumbbells are unique to our work.^11,16,35^ We did so by extracting the relative orientation between the film and individual dumbbells, enabling a precise analysis of angle distribution.

We have also highlighted the distinct characteristics of *db*_1_ and *db*_2_: *db*_1_ is ion-responsive, which can switch its film structure upon an ionic change. On the other hand, *db*_2_ exhibits ion-robustness, which maintains stable film structures under ionic changes. This can be implicated in the delivery system and emulsion stabilizers.

Future perspectives include exploring more geometrical and chemical anisotropies, such as size ratio, charge density, and polymer grafting, to manipulate a wider variety of interparticle interactions. We anticipate that this will enable innovative strategies for optimizing nanoparticle performance, increasing their applicability across industries.

## Supporting Information Available

Extended results and discussion about pH-dependent zeta potential, surface charge density, and attachment energy calculation; Experimental section; Additional data for *sp*; Full isotherm curves (PDF)
Movie S1: Wrinkle formation of nanoparticle films (MP4)
