# Supplementary material for: Regulation of Interfacial Anchoring Orientation of Anisotropic Nanodumbbells
Source: ACS Macro Lett. 2023 Sep 11;12(10):1298–305. doi: 10.1021/acsmacrolett.3c00339 (PMC10586460; doi:10.1021/acsmacrolett.3c00339)
Supplement: Supplementary file 1 — mz3c00339_si_001.pdf [file mz3c00339_si_001.pdf]

Supporting Information for

Regulation of Interfacial Anchoring Orientation of Anisotropic Nanodumbbells

Hyunwoo Jang,[†#] Chaeyeon Song,[‡#] Byungsoo Kim,[†] Chunghyeong Lee,[†] Juncheol Lee,[†] Youngkyu Han,[‡] Ilsin An,[§] Joon Heon Kim,[‖*] Jin Nam,[‡*] and Myung Chul Choi[†*]

[†]Department of Bio and Brain Engineering, Korea Advanced Institute of Science and Technology, Daejeon 34141, South Korea.

[‡]AMOREPACIFIC R&I Center, Yongin 17074, South Korea.

[§]Department of Photonics and Nanoelectronics, Hanyang University, Ansan 15588, South Korea.

[‖]Advanced Photonics Research Institute, Gwangju Institute of Science and Technology, Gwangju 61005, South Korea.

[#]These authors contributed equally.

*Corresponding authors.
Email: joonhkim@gist.ac.kr (J.H.K), apjnam@amorepacific.com (J.N.), and mcchoi@kaist.ac.kr (M.C.C.)

Extended Results and Discussion

pH-dependent zeta potential analysis

When pH is varied between 3 and 11, *sp* and db_1 showed constant ζ at approximately –60 mV (Figure S12). This is because sulfonate group has a low $pK_a \approx 1.0$.[1] Amine-modification changes the σ of seed lobe to be pH-dependent. The ζ of db_2 dramatically changes with an isoelectric pH ≈ 5.5. At high pH, db_2 becomes electrostatically equivalent to db_1 as all amine groups neutralize. We fitted the ζ curve of db_2 (light blue line in Figure S12) to the extended Henderson-Hasselbalch equation:

$$\zeta = \frac{\zeta_+}{1 + 10^{\alpha(\mathrm{pH}-\mathrm{pK_a})}} + \zeta_-$$

where ζ_+ and ζ_- are the positive and negative contributions in ζ, and α is an empirical parameter accounting for pK_a distribution. The fitted values of pK_a and α were 6.6 ± 0.6 and 0.25 ± 0.1 respectively. The obtained values of ζ_+ and ζ_- were 102 ± 26 and –67 ± 5 mV. The fitted ζ_- was close to the ζ of db_1, suggesting equal number of negative (sulfonate) groups of the two particles. With the assumption that ζ_+ and ζ_- are proportional to the number of positive and negative surface charges, this result indicates that (a) db_2 has an approximately identical amount of negative charges as db_1 and (b) the ratio of amine and sulfonate groups on db_2 is approximately 3:2. At pH 7.5, 37% of the amine groups are protonated, resulting in approximately 1:2 ratio of positive and negative charges.

Surface charge analysis

We calculated the effective surface charge values of our particles using the Grahame equation:

$$\sigma = \sqrt{8\varepsilon_0 \varepsilon k_B T\,[\mathrm{NaCl}]}\,\sinh\left(\frac{e\psi}{2k_B T}\right)$$

where ε is the relative permittivity of water, ε_0 is the permittivity of vacuum, k_B is the Boltzmann constant, T is the absolute temperature, e is the elementary charge, and ψ is the surface potential.[2] The experimentally obtained ζ values were used as ψ values. For all particles, σ was consistent within our ionic strength regime (Figure 1F). Therefore, we chose the σ values at I = 0.17 M as representative values (Table 1). To estimate the σ of seed lobe of db_1, the zeta potential of core–shell seed (before swelling) at I = 0.17 M was measured as −26.9 mV, corresponding to σ = −0.17 e nm^{-2}. This indicates approximately half of the negative charges of *sp* are masked during the coating process. The σ of db_1 budded lobe was estimated as −0.29 e nm^{-2} to yield the net σ of db_1 of −0.23 e nm^{-2}. The σ of db_2 seed lobe was estimated to be +0.03 e nm^{-2} to yield the net σ of db_2 of −0.13 e nm^{-2}, assuming that the σ of budded lobe remains unchanged at −0.29 e nm^{-2}. The net σ of db_2 being approximately half of db_1 is consistent with pH-dependent zeta potential analysis result (the ratio of positive and negative charges ≈ 1:2).

Since our aim of introducing db_2 was to use it as an experimental condition where interparticle repulsion is suppressed through chemical charge grafting, we tried to find the pH condition that minimizes interparticle repulsion without undermining suspension stability. At pH 7.5, the σ of the seed lobe is approximately neutral (+0.03 e nm^{-2}). We also found that, at pH lower than 7.5, the suspension stability decreased and particles aggregates rapidly, likely because the seed lobes become positively charged and dipole-like interparticle attraction appears. Therefore, we chose pH 7.5 as our experimental conditions.

Attachment energy calculation

We calculated the attachment energy of spherical and dumbbell PS nanoparticles (Figure S9). The attachment energy of a PS nanoparticle was defined as:

$$\Delta E = E_{\mathrm{int}} - E_{\mathrm{water}}$$

where E_{int} and E_{water} are surface free energies of the system when a particle is attached to the interface and fully submerged in water phase, respectively.[3] The two surface free energy terms are expressed as:

$$E_{\mathrm{water}} = \gamma_{\mathrm{water\text{-}PS}}\, S + \gamma_{\mathrm{air\text{-}water}}\, A$$
$$E_{\mathrm{int}} = \gamma_{\mathrm{air\text{-}PS}}\, S_{\mathrm{air}} + \gamma_{\mathrm{water\text{-}PS}}\, S_{\mathrm{water}} + \gamma_{\mathrm{air\text{-}water}}\, (A - A_{\mathrm{CS}})$$

where $\gamma_{\mathrm{a\text{-}b}}$ is the surface tension between a and b, S is the total surface area of a particle, A is the area of the air-water interface, and A_{CS} is the cross-sectional area of the particle, i.e. the decreased area of the air-water interface due to the presence of the particle. S_{air} and S_{water} are the surface areas of a particle exposed to air and water phases, respectively ($S = S_{\mathrm{air}} + S_{\mathrm{water}}$). For our calculation, the values of $\gamma_{\mathrm{air\text{-}water}}$, $\gamma_{\mathrm{air\text{-}PS}}$, and $\gamma_{\mathrm{water\text{-}PS}}$ were set to 72, 40, and 40 mJ m^{-2} respectively.[4–6] Especially, $\gamma_{\mathrm{air\text{-}PS}}$ and $\gamma_{\mathrm{water\text{-}PS}}$ were set equally, based on the

experimental results showing that the contact angle of air, water, and PS is close to 90°.[4] This renders the energy landscape symmetric. For dumbbell particles, we set the surface tension of two lobes as equal, assuming that the surface tension of polystyrene remains consistent under surface charge variations.[7] Then ΔE can be simplified as:

$$\Delta E = -\gamma_{\text{air-water}} A_{\text{CS}}$$

Figure S9A,B show that sphere and dumbbell PS nanoparticles anchored at the middle of the interface have the adhesion energy barriers of $2.4 \times 10^5 k_B T$ (sphere) and $7.3 \times 10^5 k_B T$ (dumbbell) to be detached to either phase. Figure S9C shows that a dumbbell particle (anchored at the middle) has the rotational energy barrier of $5.5 \times 10^5 k_B T$ to be rotated from horizontal to vertical orientation.

Experimental Section

Synthesis of dumbbell nanoparticles

Seed particles (i.e., *sp*) were radical-polymerized from styrene monomers with sodium 4-vinylbenzenesulfonate (1% w/v) as a stabilizer and potassium persulfate as an initiator (0.1% w/v). The db_1 was bulk-synthesized using a two-step seeded emulsion technique.[8] First, *sp*–poly(styrene-*co*-TMSPA) core–shell nanospheres (*CS*) were synthesized through radical polymerization in water condition. The *sp* suspension (10.5% w/v) was mixed with styrene, 3-(trimethoxysilyl) propyl acrylate (TMSPA, 10.5% v/v), and an initiator (AIBN, azobisisobutyronitrile, 0.5% w/v) and reacted for 8 h at 70°C. The volume ratio of the *sp* suspension and St/TMSPA monomer solution was kept at 1.0. Second, the *CS* suspension (10.0% w/v) was mixed for 8 h at 70°C with a mixture of swelling styrene monomers and AIBN (0.5% w/v). The volume ratio between the *CS* suspension and the monomer solution was fixed at 1.1, resulting in a precisely symmetric dumbbell morphology. A small amount (0.05% v/v) of nonionic surfactant Synperonic® F 108 (Sigma-Aldrich) was added to increase the dispersion of the *CS*, which is critical for the size uniformity of db_1. The resultant db_1 suspension was washed by ultra-centrifugation and redispersion in DI water at least three times to remove unreacted residuals and impurities.

The db_2 was synthesized by amine-modifying the seed lobe of db_1 by silane coupling.[9] As a silane coupling agent, 0.5 mL *N*-[3-(trimethoxysilyl)propyl] ethylenediamine (TMSPE) and 1.0 mL of 30% ammonium hydroxide (NH_4OH, Sigma-Aldrich) solution was added to 1.0 mL db_1 suspension (1.5% w/v in ethanol). The silane groups on the db_1 seed lobe surface reacted with the coupling agent for 24 h at room temperature. Excess coupling agent and NH_4OH were removed from the aqueous phase by repeated centrifugation and redispersion in DI water. All NP suspensions were stored at 4°C.

Particle morphology (size and aspect ratio) were verified with SEM (Figure S13). The radius and length differences of db_1 and db_2 were statistically insignificant. NP suspensions were extensively diluted and spread on gold plates. The gold plates were dried in an oven at 60°C and imaged with SEM. The electrochemical profiles of db_1 and db_2 were also verified by AuNP adsorption. The particle suspension was overnight mixed with 20 nm anionic AuNPs (capped with citrate) or cationic AuNPs (capped with

cysteamine for db_1 and branched polyethylenimine [BPEI] for db_2) at 1:20 number ratio of nano-dumbbells and AuNPs.

Zeta potential measurement

NP suspension was diluted to 0.001% w/v with a water phase of a specific ionic strength. Then the mixture was sonicated for >15 min. Zeta potential was measured using a Zetasizer ZS90 (Malvern Instruments).

Pair interaction energy calculation

We approximated the interfacial particle interaction as the interaction in the bulk aqueous condition, based on the finding that the interaction between charged colloidal particles at the air-water interface is very close to bulk condition for 1:1 electrolyte.[10] We simplified the dumbbells as two interconnected spheres with the radii and charge densities in Table 1. Based on the high attachment energy ($7.3 \times 10^5 k_BT$, Figure S9), we constrained that two dumbbells are anchored at the middle of the interface, horizontally[11].

The van der Waals potential between two spheres is given as $V_{vdW} = -A_HR/12D$ where A_H is the Hamaker constant (3.163 k_BT for the polystyrene-water-polystyrene interaction),[12] R is the radius of the sphere, and D is the surface-to-surface distance between two spheres. The electrostatic potential term of two charged spheres is given as $V_{el} = 2\pi R\sigma_1\sigma_2 e^{-\kappa D}/\varepsilon\varepsilon_0\kappa^2$, where ε is the relative permittivity of water, ε_0 is the permittivity of vacuum, and κ is the reciprocal of the Debye length. For σ_1 and σ_2, the charge density values in Table 1 were used.

For dumbbell particles, the interaction energy varies depending on the approaching directions (θ) and relative orientations (φ). The interaction energy versus surface-to-surface distance was calculated for every angle combination of θ and φ with a 1° interval. We placed both dumbbells at the center, displaced the second dumbbell by 1 μm in a certain direction (θ) while fixing the first dumbbell at the center. We then rotated the second dumbbell on the interfacial plane by a certain angle (φ), and then brought the second dumbbell toward the center until the surface-to-surface distance between the two dumbbells becomes zero. Refer to Figure S14 for the schematics. See Figures S2 and S3 for complete, unaveraged curves.

The primary geometrical difference of sphere and dumbbell is that dumbbells can establish multiple contact point. In our calculation at 0.17 M (where equilibrium spacing is present), the energy well at the secondary minimum of db_1 deepened as the number of contact points increased (Figure S14B,C). This suggests that db_1 particles initially weakly-attached at single contact point may rotate to maximize the number of contact points.

Foam formation

The ionic strength of the water phase was adjusted by either adding NaCl to or diluting 1x PBS. NP suspensions were diluted with an aqueous phase to yield 0.2% (w/v). To obtain representative photographs, 5 mL of the NP suspensions were vortexed for 1 min and hand-shaken for 2 min.[13] To

quantify the foam height, 1.0 mL of NP suspension was inserted into a glass tube (outer diameter = 5 mm, length = 18 cm) and hand-shaken for 2 min. The foam height was measured after 10 min.

For the defoaming experiment, 0.5 mL of the high salt (I = 1.0 M) NP suspension was inserted into a glass tube and hand-shaken for 2 min. After 10 min, the foam height was measured and 0.25 mL of DI water was inserted into the tube. The salt dilution and measurement were repeated six times.

For cryo-SEM imaging, a foam sample was placed on a cryo plate and deep-frozen with liquid nitrogen. Samples were sublimated for 5 min and Pt-coated in a cryo-preparation chamber (Quorum P3010T). Samples were fractured with a razor blade and imaged with FIB-SEM (Zeiss Crossbeam 550) located at Korea Research Institute of Chemical Technology.

Nanoparticle deposition and compression

The NP suspension (~10%, w/v) was diluted with isopropanol (IPA, >99.9%, electronic grade; 1:3, v/v).[13,14] The NP-IPA mixture was sonicated for >15 min and spread dropwise on the subphase contained in a Teflon trough on top of a vibration isolation table. Before subsequent experiments, the IPA was allowed to evaporate for >15 min. In all experiments, the NPs were laterally and bidirectionally compressed by two Delrin barriers at the speed of 4.2 mm min^{-1} (trough area decreases by 3.4 cm^2 min^{-1}).

Cross-sectional SEM imaging

Si wafers were hydrophobic-coated with octadecyltrichlorosilane (OTS, Sigma-Aldrich).[15,16] Briefly, piranha-cleansed Si wafers (15×10 mm) were exposed to 254-nm UV light (2 mW cm^{-2}) for 1 h and immersed in 1 mM OTS solution in isooctane (>98%) for 1 h. The coated wafers were rinsed with isooctane at least three times and desiccated overnight. The NP films were transferred onto the Si wafers using the Langmuir-Schaefer (LS) method.[15] After reaching π_c, an OTS-coated wafer was lowered downward at the speed of 2 mm min^{-1}. After touching the NP film, the wafer was retracted at the same speed and desiccated overnight. SEM images were taken at the fractured area of the NP film, using a Zeiss GeminiSEM 300 in the AMOREPACIFIC R&I Center.

In-situ gel trapping

A solution of photoinitiator 2,2-Dimethoxy-2-phenylacetophenone (DMPA, Sigma-Aldrich) was prepared by dissolving 0.3 g of DMPA in 2 mL of ethanol. This DMPA solution was subsequently mixed with 150 mL of the 1x PBS subphase. Excess DMPA precipitate was separated by centrifugation. Then the mixture was poured to the Langmuir trough and db_1 particles were deposited on the subphase. After compressing the db_1 film on 1x PBS up to π_c, acrylamide/bis-acrylamide was gradually introduced to the subphase.

For this, a 75 mL solution of 40% acrylamide/bis-acrylamide (19:1) in water was prepared and its ionic strength was set to 0.17 M by adding NaCl. A 3 mL of the subphase was gently extracted from the trough and mixed with the 75 mL acrylamide/bis-acrylamide solution. Then the trough was refilled with 3 mL of that solution. This exchange process was repeated 60 times. During this procedure, the subphase in the trough was gently stirred at 100 rpm to ensure homogeneous mixing of acrylamide/bis-acrylamide without disrupting the particle film. As a result, the final concentration of acrylamide/bis-acrylamide in

the subphase reached ~13%. Although ethanol is introduced to the subphase, given the small volume ratio of ethanol to water (1:112.5), its effect on interparticle forces is expected to be minimal.

For curing, 312-nm UV light (1 mW cm^{-2}) was applied for 15 min. As a result, NPs were trapped in the subphase gel while maintaining their ordering structure. Samples were imaged with FIB-SEM (Zeiss Crossbeam 550) located at the Korea Research Institute of Chemical Technology.

Orientation angle (OA) measurement

Based on cross-sectional SEM images, we determined the 3D spherical coordinates of the normal vector of the ANP film plane and the dumbbell vector (pointing toward the long axis) and calculated the angle between the two vectors. The angles describing the normal vector of the ANP film plane (θ_p and φ_p) were estimated from the average area of each lobe displayed in the image and the 2D angle between a straight line encompassing the cross section and the horizontal axis of the image. The angles describing the dumbbell vectors (θ_d and φ_d) were estimated from the apparent distance between the center of two lobes on the image and the 2D angle between the long axis and the horizontal axis of the image.

The image plane is defined as the y-z plane (x-axis perpendicular to the image pointing outwards). For each SEM image, we determined the orientation of the normal unit vector $\mathbf{n_p}$ of the ANP film plane (orientation defined by θ_p and φ_p; See Figure S7A,E for details on notations). First, a straight line was drawn along the cross section of the film. Then, another line perpendicular to the first line was drawn. The direction of this second line corresponded to the direction of $\mathbf{n_p}$ projected onto the y-z plane (denoted as $\mathbf{n_p^{proj}}$). Note that the length of $\mathbf{n_p^{proj}}$ is equal or smaller than one. The angle between $\mathbf{n_p^{proj}}$ and y-axis was defined as α_p. Second, the average area of each lobe displayed in the image (A') divided by the average area of lobes in the top-view image ($A = 0.02\ \mu\text{m}^2$) was defined as k (Figure S7C). Then the followings hold:

$$k = \sin\theta_p \cos\varphi_p$$
$$\tan\alpha_p = \frac{\cos\theta_p}{\sin\varphi_p \sin\theta_p}$$

Then with respect to φ_p, the followings hold:

$$\cos^2\varphi_p + \sin^2\varphi_p = \frac{k^2}{\sin^2\theta_p} + \frac{\cos^2\theta_p}{\tan^2\alpha_p \sin^2\theta_p} = \frac{k^2\tan^2\alpha_p + (1 - \sin^2\theta_p)}{\tan^2\alpha_p \sin^2\theta_p} = 1$$
$$\sin^2\theta_p = \frac{k^2\tan^2\alpha_p + 1}{\tan^2\alpha_p + 1} = k^2\sin^2\alpha_p + \cos^2\alpha_p$$

Thus, θ_p and φ_p can be calculated as follows:

$$\theta_p = \sin^{-1}\left(\sqrt{k^2\sin^2\alpha_p + \cos^2\alpha_p}\right)$$

$$\varphi_{\mathrm{p}} = \cos^{-1}\left(\frac{k}{\sin\theta_{\mathrm{p}}}\right) = \cos^{-1}\left(\frac{k}{\sqrt{k^2\sin^2\alpha_{\mathrm{p}} + \cos^2\alpha_{\mathrm{p}}}}\right)$$

Next, we measured the orientation of the dumbbells. The dumbbell vector $\mathbf{v_d}$ is defined as a unit vector starting from the dumbbell's center of mass and pointing toward the center of the front lobe (a lobe shown in front of the other lobe within the image; See Figure S7B,F for details on notations). Within the SEM image, $\mathbf{v_d}$ is projected onto the *y-z* plane; also denoted as $\mathbf{v_d^{proj}}$. The two angles of $\mathbf{v_d}$ in the spherical coordinate (θ_d and φ_d) are determined as follows. First, the coordinates of the centers of two lobes were measured. Next, we obtained $m \equiv d'/d$, where d is the actual center-to-center distance of two lobes (127 nm for db_1, 123 nm for db_2) and d' is the apparent center-to-center distance measured from the image (d projected to *y-z* plane; Figure S7D). Then, the angle between the line connecting the two centers and the horizontal axis (y-axis) is denoted as α_d (Figure S7D). Then the followings hold:

$$\frac{d'}{d} \equiv m = \left\|\mathbf{v_d^{proj}}\right\| = \sqrt{\sin^2\varphi_{\mathrm{d}}\sin^2\theta_{\mathrm{d}} + \cos^2\theta_{\mathrm{d}}}$$
$$\sin\alpha_{\mathrm{d}} = \frac{\cos\theta_{\mathrm{d}}}{\left\|\mathbf{v_d^{proj}}\right\|} = \frac{\cos\theta_{\mathrm{d}}}{m}$$
$$\cos\alpha_{\mathrm{d}} = \frac{\sin\varphi_{\mathrm{d}}\sin\theta_{\mathrm{d}}}{\left\|\mathbf{v_d^{proj}}\right\|} = \frac{\sin\varphi_{\mathrm{d}}\sin\theta_{\mathrm{d}}}{\mathrm{m}}$$

Thus, θ_d and φ_d can be obtained as follows:

$$\theta_{\mathrm{d}} = \cos^{-1}(m\sin\alpha_{\mathrm{d}})$$
$$\varphi_{\mathrm{d}} = \sin^{-1}\left(\frac{m\cos\alpha_{\mathrm{d}}}{\sin\theta_{\mathrm{d}}}\right) = \sin^{-1}\left(\frac{m\cos\alpha_{\mathrm{d}}}{\sqrt{1 - m^2\sin^2\alpha_{\mathrm{d}}}}\right)$$

Now that we know the spherical coordinates of the unit vectors $\mathbf{n_p}$ and $\mathbf{v_d}$, we can calculate the angle between $\mathbf{n_p}$ and $\mathbf{v_d}$ by calculating their inner product. The orientation angle (OA) of the dumbbell was obtained as $\left|90^\circ - \cos^{-1}(\mathbf{n_p}\cdot\mathbf{v_d})\right|$ (Figure S7G). The validity of our measurement method was confirmed using model dumbbell particle images with known rotation angles (Figure S8).

Adsorption affinity measurement

After spreading particles on the subphase, total of 3 mL of subphase was sampled from 30 different loci (0.1 mL of subphase extracted for each sampling) and was sonicated for >15 min. Its turbidity (absorbance at 310 nm) was measured using a NanoDrop 2000C (Thermo Fisher Scientific Inc.). The concentration of NPs in the sample subphase was estimated from the standard curve. The number of submerged NPs was calculated by multiplying the total subphase volume and the concentration of NPs in the sample subphase. The number of NPs adsorbed on the interface was estimated by subtracting the number of submerged NPs from the total number of deposited NPs.

π-A isotherm measurement

Langmuir balance module of 1232D1-Shuttle (Nima Technology Ltd.) was used. A paper Wilhelmy plate was installed in the middle of the trough parallel to the direction of compression. The raw area per particle (A_p) was rescaled with adsorption affinity as follows:

$$A_p = \frac{\text{(trough area)}}{\text{(number of deposited NPs)} \times \text{(ratio of adsorbed NPs)}} = \frac{\text{(trough area)}}{\text{(number of adsorbed NPs)}}$$

See Figure S11 for isotherm curves before rescaling.

Optical imaging

An inverted-type optical microscope (IX-71, Olympus) with 10x magnification (UPLFLN10X, NA = 0.30, WD = 10 mm) was used. For DFM and BFM imaging, a dry dark-field condenser (U-DCD) and a bright-field condenser (IX2-LWUCD) were used respectively. For wrinkle phase images, the images were binarized and autocorrelation was calculated for all horizontal lines. Representative 1D autocorrelation curves were obtained by averaging the autocorrelation curves of all horizontal lines.

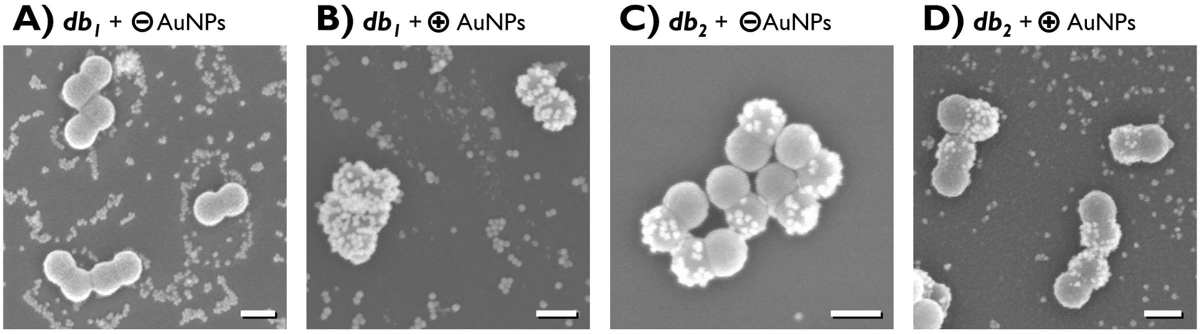

Figure S1. Charge profiles of db_1 and db_2 assessed by AuNP adsorption. (**A**) Negatively charged AuNPs showing no adsorption on both lobes of db_1. (**B**) Positively charged AuNPs adsorbed on both lobes of db_1. (**C**) Negatively charged AuNPs adsorbed on amine-modified seed lobes of db_2. (**D**) Positively charged AuNPs adsorbed on budded lobes of db_2. Experiments conducted at DI water (pH ≈ 6). Scale bars: 200 nm.

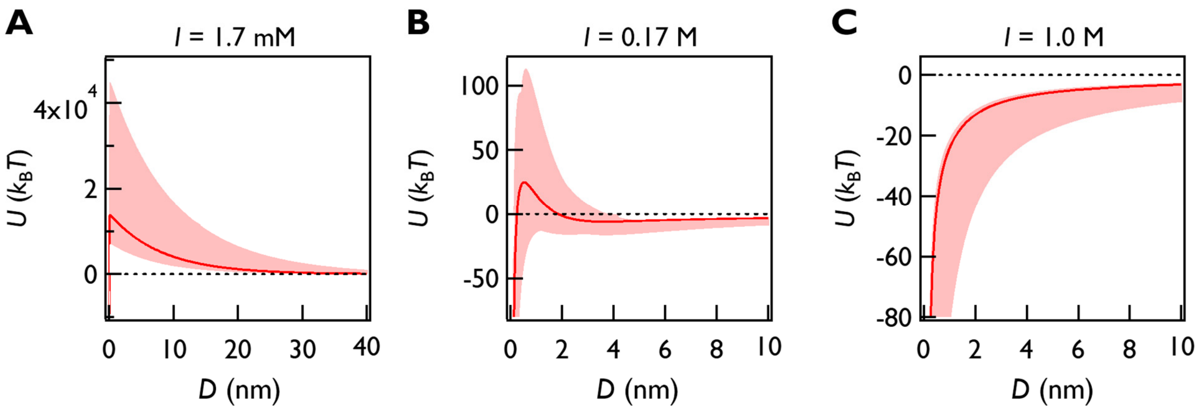

Figure S2. DLVO interaction energy of db_1 at I = (**A**) 1.7 mM, (**B**) 0.17 M, and (**C**) 1.0 M. Light red area: entire range of interaction energy for all angle combinations. Red lines: average curves.

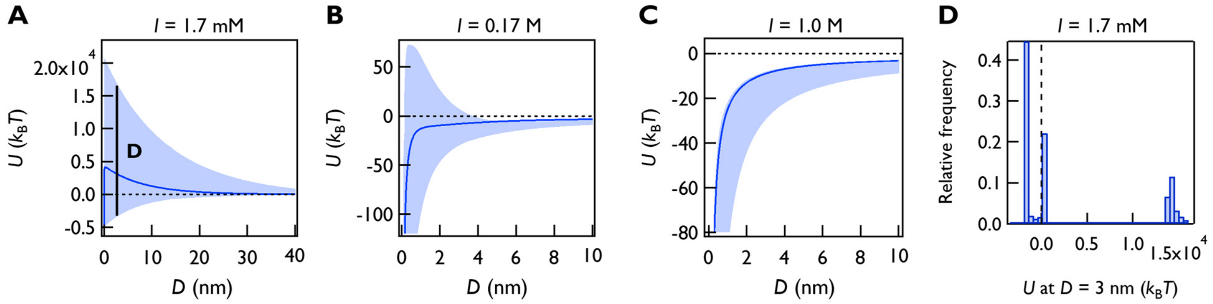

Figure S3. DLVO interaction energy of db_2 at I = (**A**) 1.7 mM, (**B**) 0.17 M, and (**C**) 1.0 M. Light blue area: entire range of interaction energy in all angle combinations. Blue lines: average curves. In (A), black line denotes D = 3 nm. (**D**) Histogram of interaction energy at D = 3 nm for I = 1.7 mM in all angle combinations.

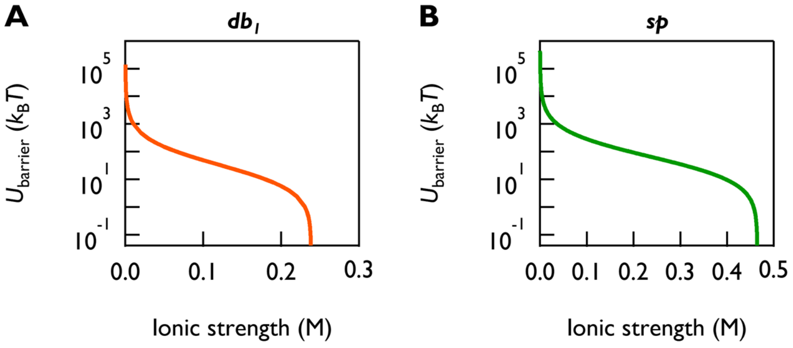

Figure S4. $U_{barrier}$ of (**A**) db_1 and (**B**) *sp* as a function of ionic strength. $U_{barrier}$ reaches zero at I = 0.24 M and 0.46 M for db_1 and *sp*, respectively.

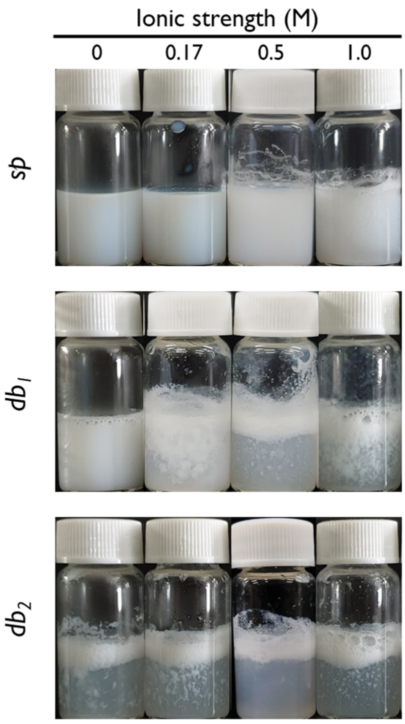

Figure S5. Photographs of NP-stabilized foams at various ionic strength. Images were taken 1 h after foaming.

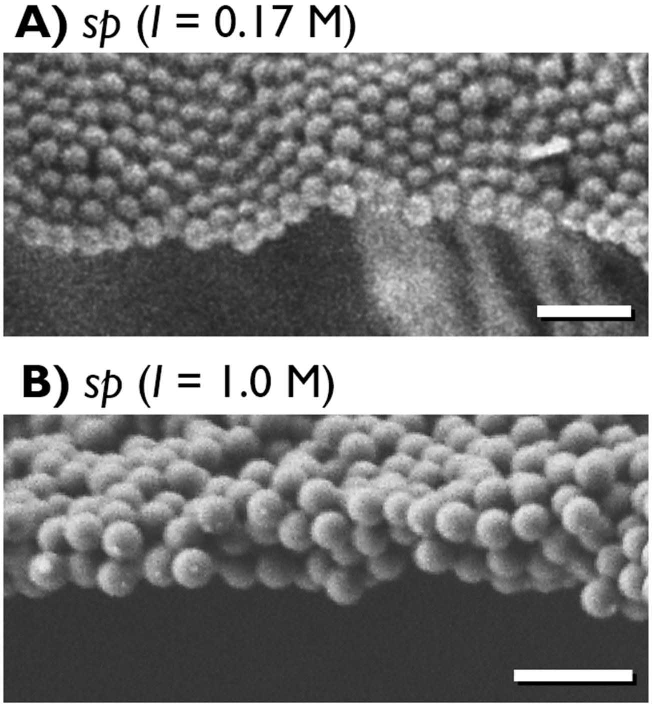

Figure S6. Cross-sectional SEM images of *sp* films at I = (**A**) 0.17 M and (**B**) 1.0 M. Scale bars: 200 nm.

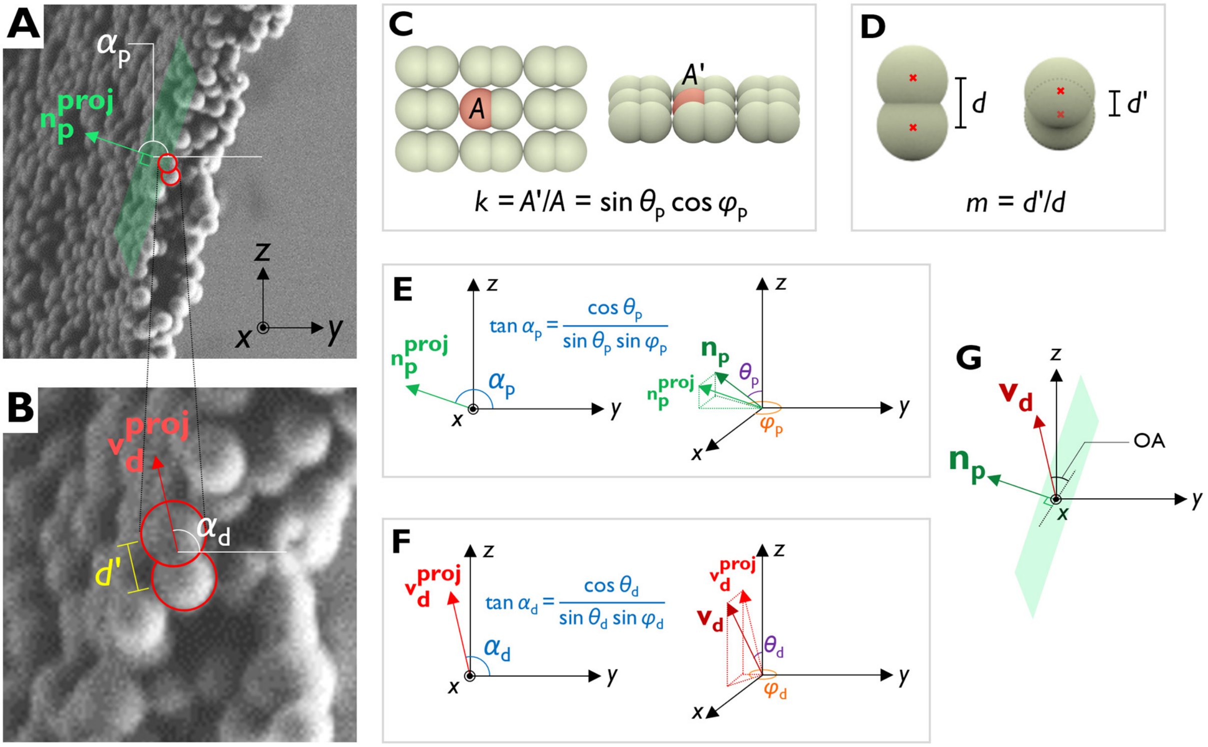

Figure S7. Notations for orientation angle calculation. (**A**) An example SEM image showing normal vector of the ANP plane ($\mathbf{n_p}$) projected on the *y-z* plane (denoted as $\mathbf{n_p^{proj}}$**).** The angle between $\mathbf{n_p^{proj}}$ and horizontal line (*y*-axis) defined as α_p. One dumbbell is highlighted with red circles. (**B**) Zoom-in view of (A), showing a dumbbell vector ($\mathbf{v_d}$) projected on the *y-z* plane (denoted as $\mathbf{v_d^{proj}}$). The angle between $\mathbf{v_d^{proj}}$ and horizontal line (*y*-axis) defined as α_d. Apparent distance between the centers of two lobes denoted *d'*. (**C**) Schematics defining *A*, *A'*, and *k*. (**D**) Schematics defining *d*, *d'*, and *m*. (**E**) Schematics of $\mathbf{n_p}$ viewed from two perspectives. Two angles defining the direction of $\mathbf{n_p}$ (θ_p and φ_p) are depicted. (**F**) Schematics of $\mathbf{v_d}$ viewed from two perspectives. Two angles defining the direction of $\mathbf{v_d}$ (θ_d and φ_d) are depicted. (**G**) Orientation angle (OA) defined as $|90° - (\text{angle between } n_p \text{ and } v_d)|$.

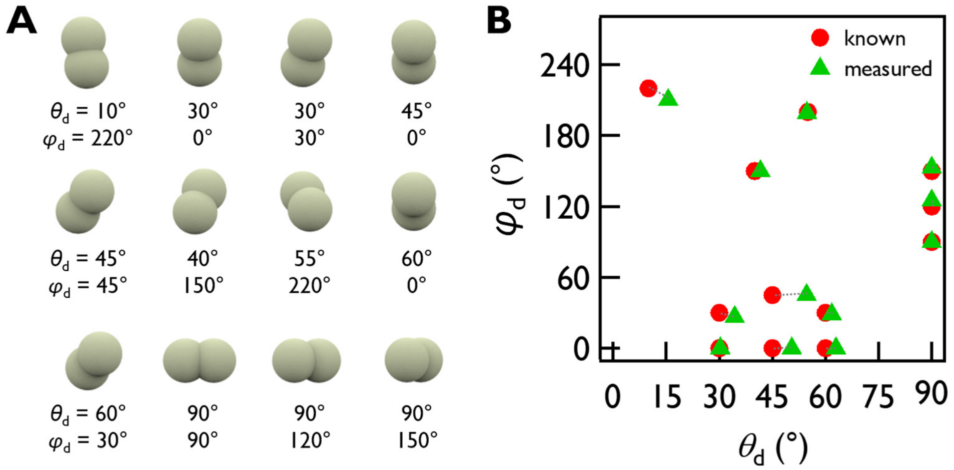

Figure S8. (**A**) Dumbbell particle model images with known rotation angle values. (**B**) The angles of dumbbell particles (θ_d and φ_d) measured from the images in (A). The pairs of known values and measured values (using our analysis) connected with gray lines.

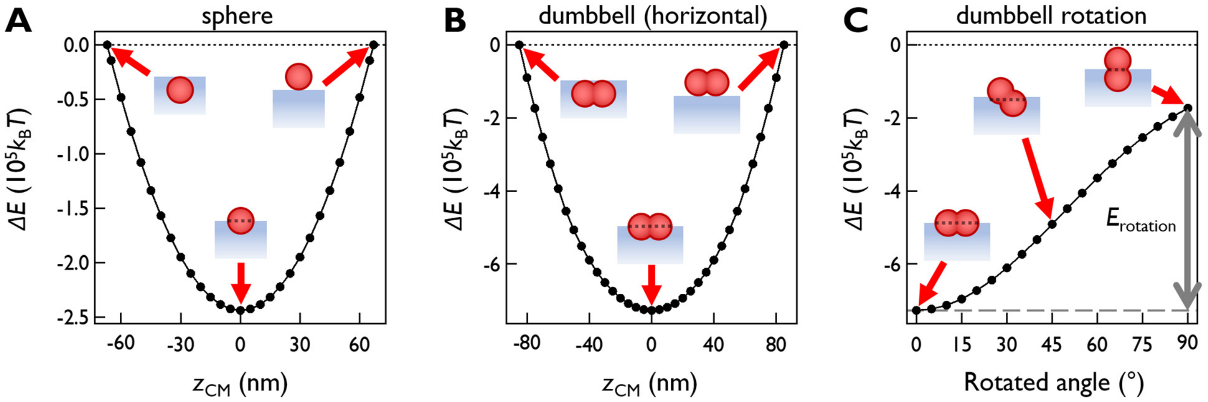

Figure S9. Attachment energy of polystyrene nanoparticles vs. z position and rotation angle. (**A**) Attachment energy of polystyrene sphere (R = 67 nm) vs. z-coordinate of particle's center of mass (z_{CM}) relative to the air-water interface. (**B**) Attachment energy of horizontally oriented polystyrene dumbbell (R = 85 nm, L = 297 nm) vs. z_{CM}. (**C**) Attachment energy of polystyrene dumbbell vs. rotated angle. Horizontal and vertical orientations correspond to 0° and 90°, respectively. Rotational energy barrier ($E_{rotation}$) corresponds to attachment energy difference between 0° and 90°.

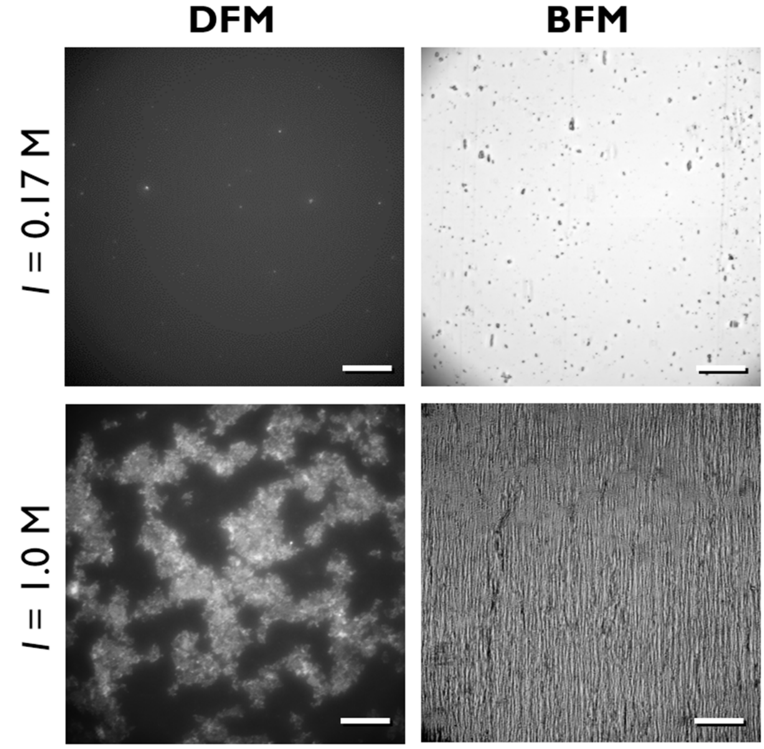

Figure S10. DFM and BFM images of *sp* at I = 0.17 M and 1.0 M. DFM images were obtained before lateral compression (π = 0 mN m^{-1}). BFM images were obtained at collapse pressure. See also Movie S1. Scale bars: 200 μm.

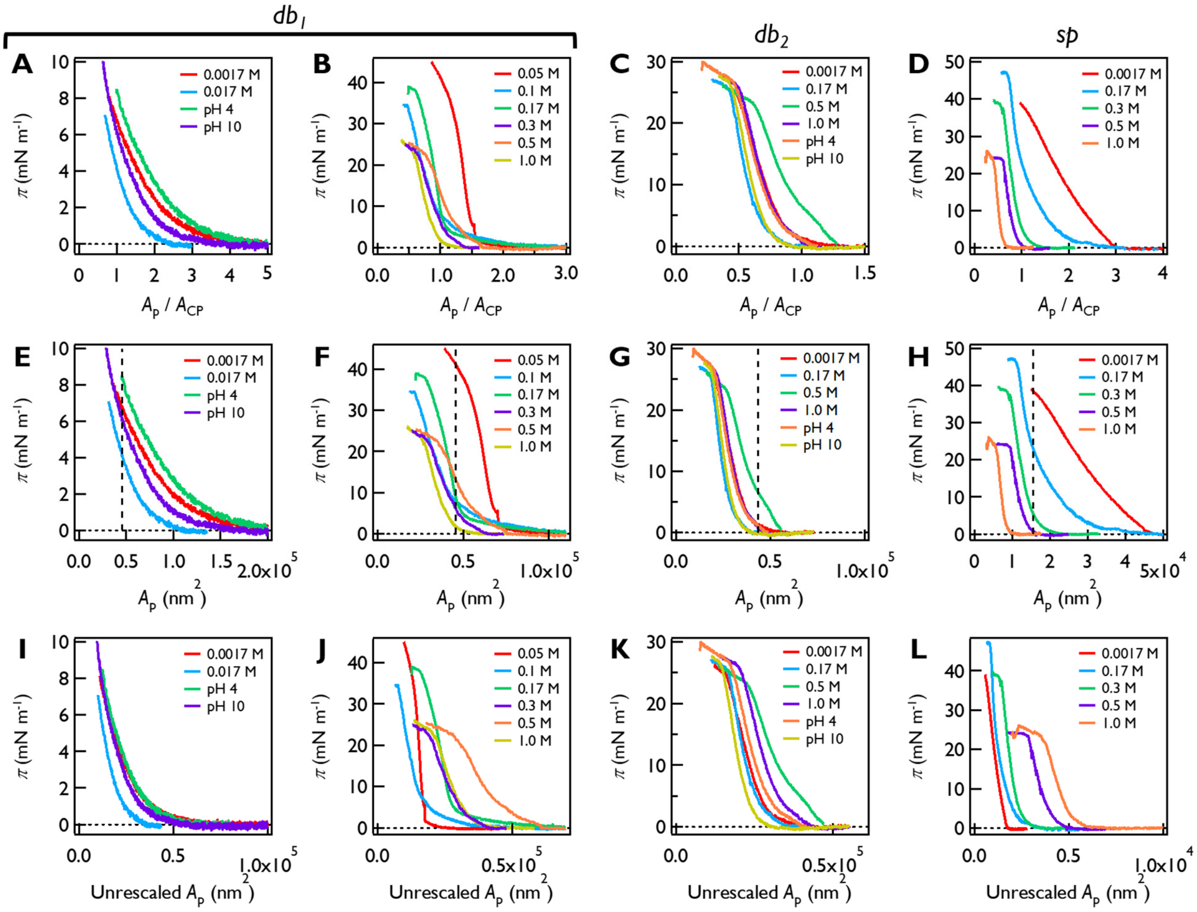

Figure S11. (**A**-**D**) Full isotherm data of the three NPs. (**E**-**H**) Isotherm graphs without normalization with A_{CP}. Vertical dashes: A_{CP}. (**I**-**L**) Raw isotherm graphs before rescaling with adsorption affinity.

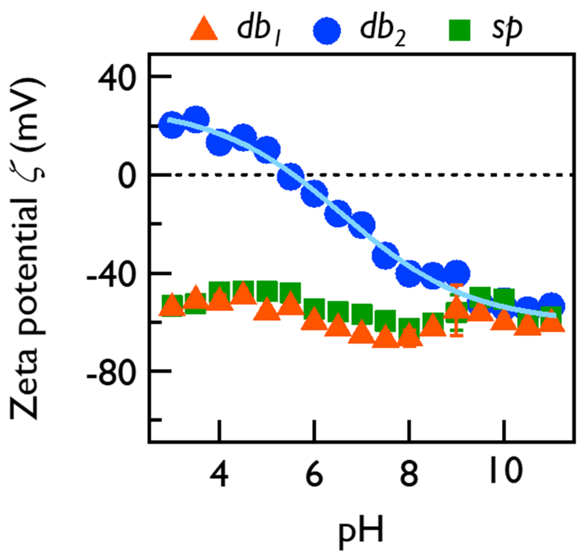

Figure S12. Zeta potential of the three NPs as a function of pH. Ionic strength kept below 2 mM. Solid line: fit to Henderson-Hasselbalch equation.

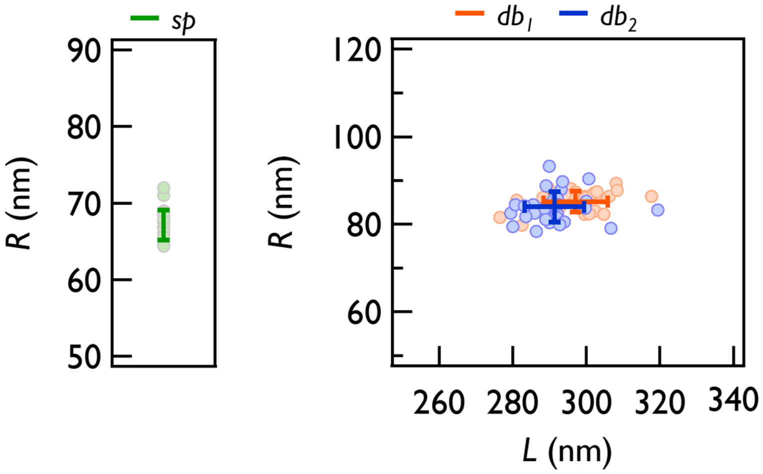

Figure S13. Size distributions of (**A**) *sp*, (**B**) db_1, and db_2. *R*: radius of host sphere. *L*: length to long axis. Error bars: standard deviations.

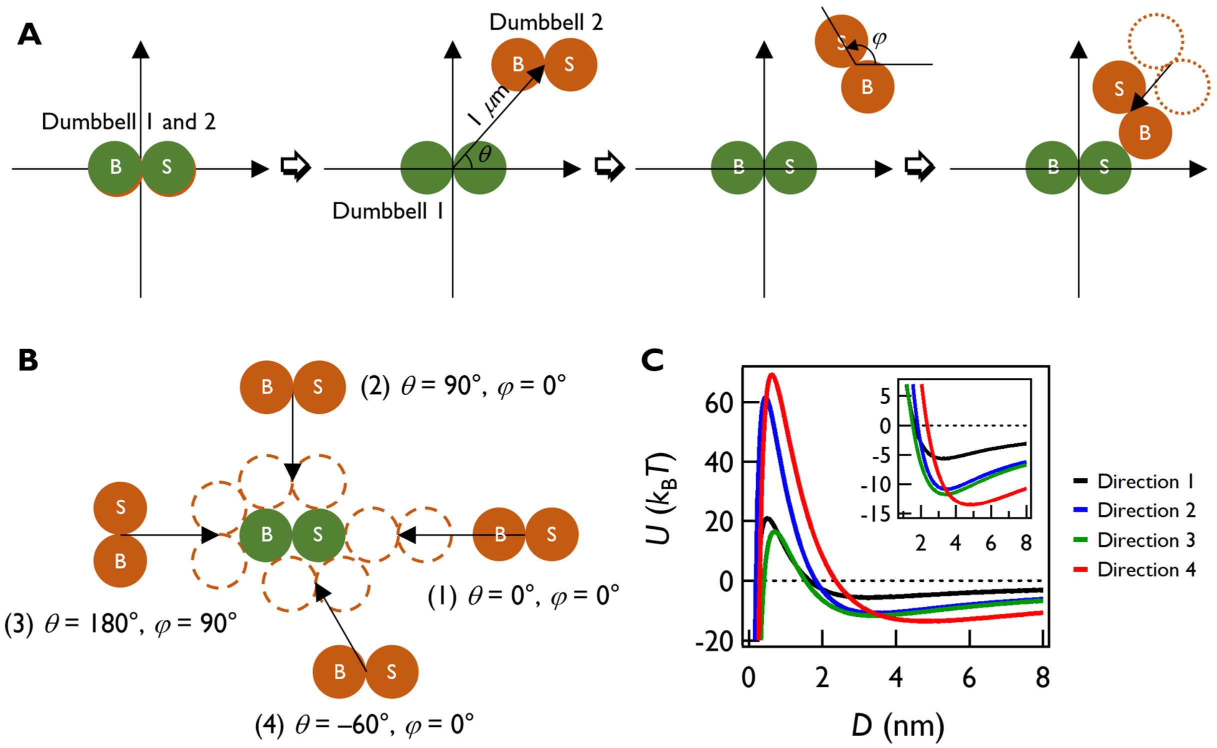

Figure S14. Direction-dependent interaction energy of two dumbbell particles. (**A**) Schematics of energy calculation procedures. Seed lobes and budded lobes indicated as S and B. Schematics are not in scale. (**B**) Four representative approaching directions. Directions 2 and 3 result in two contact points. Direction 4 results in three contact points. (**C**) Pair interaction energy of db_1 at I = 0.17 M for the four approaching directions. Direction 4 (red) exhibits the highest U_{barrier} and the deepest energy well at the secondary minimum.

SI References

(1) Lewis, S. R.; Datta, S.; Gui, M.; Coker, E. L.; Huggins, F. E.; Daunert, S.; Bachas, L.; Bhattacharyya, D. Reactive Nanostructured Membranes for Water Purification. *Proceedings of the National Academy of Sciences* **2011**, *108* (21), 8577–8582. https://doi.org/10.1073/pnas.1101144108.

(2) Israelachvili, J. N. *Intermolecular and Surface Forces*, 3rd Edition.; Elsevier, 2011. https://doi.org/10.1016/C2009-0-21560-1.

(3) Park, B. J.; Lee, D. Equilibrium Orientation of Nonspherical Janus Particles at Fluid-Fluid Interfaces. *ACS Nano* **2012**, *6* (1), 782–790. https://doi.org/10.1021/nn204261w.

(4) Li, Y.; Pham, J. Q.; Johnston, K. P.; Green, P. F. Contact Angle of Water on Polystyrene Thin Films: Effects of CO2 Environment and Film Thickness. *Langmuir* **2007**, *23* (19), 9785–9793. https://doi.org/10.1021/la0636311.

(5) Lee, L. -H. Relationships between Surface Wettability and Glass Temperatures of High Polymers. *J Appl Polym Sci* **1968**, *12* (4), 719–730. https://doi.org/10.1002/app.1968.070120410.

(6) CHO, J. S.; HAN, S.; KIM, K. H.; HAN, Y. G.; LEE, J. H.; LEE, C. S.; SUNG, J. W.; BEAG, Y. W.; KOH, S. K. Surface Modification of Polymers by Ion-Assisted Reactions: An Overview. In *Adhesion Aspects of Thin Films*; Mittal, K., Ed.; 2005; Vol. 2, pp 105–121.

(7) Zheng, R.; Binks, B. P. Pickering Emulsions Stabilized by Polystyrene Particles Possessing Different Surface Groups. *Langmuir* **2022**, *38* (3), 1079–1089. https://doi.org/10.1021/acs.langmuir.1c02648.

(8) Park, J.-G.; Forster, J. D.; Dufresne, E. R. High-Yield Synthesis of Monodisperse Dumbbell-Shaped Polymer Nanoparticles. *J Am Chem Soc* **2010**, *132* (17), 5960–5961. https://doi.org/10.1021/ja101760q.

(9) Kim, J. W.; Lee, D.; Shum, H. C.; Weitz, D. A. Colloid Surfactants for Emulsion Stabilization. *Advanced Materials* **2008**, *20* (17), 3239–3243. https://doi.org/10.1002/adma.200800484.

(10) Girotto, M.; Dos Santos, A. P.; Levin, Y. Interaction of Charged Colloidal Particles at the Air-Water Interface. *Journal of Physical Chemistry B* **2016**, *120* (26), 5817–5822. https://doi.org/10.1021/acs.jpcb.5b10105.

(11) Ballard, N.; Bon, S. A. F. Equilibrium Orientations of Non-Spherical and Chemically Anisotropic Particles at Liquid–Liquid Interfaces and the Effect on Emulsion Stability. *J Colloid Interface Sci* **2015**, *448*, 533–544. https://doi.org/10.1016/j.jcis.2015.02.069.

(12) Israelachvili, J. *Intermolecular and Surface Forces*; Academic Press, 2011. https://doi.org/10.1016/C2009-0-21560-1.

(13) Hunter, T. N.; Jameson, G. J.; Wanless, E. J.; Dupin, D.; Armes, S. P. Adsorption of Submicrometer-Sized Cationic Sterically Stabilized Polystyrene Latex at the Air–Water Interface: Contact Angle Determination by Ellipsometry. *Langmuir* **2009**, *25* (6), 3440–3449. https://doi.org/10.1021/la803879p.

(14) Lenis, J.; Razavi, S.; Cao, K. D.; Lin, B.; Lee, K. Y. C.; Tu, R. S.; Kretzschmar, I. Mechanical Stability of Polystyrene and Janus Particle Monolayers at the Air/Water Interface. *J Am Chem Soc* **2015**, *137* (49), 15370–15373. https://doi.org/10.1021/jacs.5b10183.

(15) Wen, T.; Majetich, S. A. Ultra-Large-Area Self-Assembled Monolayers of Nanoparticles. *ACS Nano* **2011**, *5* (11), 8868–8876. https://doi.org/10.1021/nn2037048.

(16) Khatri, O. P.; Biswas, S. K. Friction of Octadecyltrichlorosilane Monolayer Self-Assembled on Silicon Wafer in 0% Relative Humidity. *The Journal of Physical Chemistry C* **2007**, *111* (6), 2696–2701. https://doi.org/10.1021/jp067206s.
